# Applications of chitosan (CHI)-reduced graphene oxide (rGO)-polyaniline (PAni) conducting composite electrode for energy generation in glucose biofuel cell

**DOI:** 10.1038/s41598-020-67253-6

**Published:** 2020-06-26

**Authors:** Sufia ul Haque, Abu Nasar, Inamuddin, Mohammed Muzibur Rahman

**Affiliations:** 1 Advanced Functional Materials Laboratory, Department of Applied Chemistry, Faculty of Engineering and Technology, Aligarh Muslim University, Aligarh, 202002 India; 2 Chemistry Department, Faculty of Science, King Abdulaziz University, P. O. Box 80203, Jeddah, 21589 Saudi Arabia

**Keywords:** Energy, Chemistry, Energy science and technology, Materials science

## Abstract

A glassy carbon electrode (GC) immobilized with chitosan (CHI)@reduced graphene (rGO)-polyaniline (PAni)/ferritin (Frt)/glucose oxidase (GOx) bioelectrode was prepared. The prepared electrode was characterized by using cyclic voltammetry (CV), linear sweep voltammetry (LSV) and electrochemical impedance spectroscopy (EIS) techniques. The morphological characterization was made by scanning electron microsopy (SEM) and Fourier transform infrared (FTIR) spectroscopy. This bioelectrode provided a stable current response of 3.5 ± 0.02 mAcm^−2^ in 20 mM glucose. The coverage of enzyme on 0.07 cm^2^ area of electrode modified with CHI@rGO-PAni/Frt was calculated to be 3.80 × 10^−8^ mol cm^−2^.

## Introduction

The pursuit of sustainable and green energy sources emerges because of the uneven geographical dispersal of fossil fuels, which are linked with the severe effects of environmental pollution. In this context, fuel cells have emerged as the eco-friendly electrochemical devices that directly convert the chemical energy of fuel into electrical energy^1^. Generally, conventional fuel cells utilized noble metals like Pt, Ru, Pd, and their alloys as catalysts for the conversion of fuel at the anodic chamber and the reduction of oxidant at the cathodic chamber. However, the noble metal catalysts are expensive and depleteable resources which only exist at limited places on the earth. Additionally, the electrolytes used at higher pH require expensive membranes to isolate the chemical reactions into an individual chamber, which poses one more challenge.

Moreover, renewable and clean energy is required in biomedical implantable and portable electronic devices^2,3^. The range of potential applications of biofuel cells are microelectronics, sensors and biomedical devices^4,5^. But insufficient durability and biocompatibility raise a concern about health and safety, which poses substantial challenges in the productive growth of such devices. Enzymatic biofuel cells (EFCs) are one of the subsets of fuel cells utilizing enzymes as catalysts^6–12^. The applications of enzyme bioelectrocatalysts for the conversion of chemical energy into electrical energy have several benefits. First of all, the catalyst used is renewable as the redox enzymes can be taken out from a wide variety of living organisms. Secondly, a variety of fuels can be utilized such as sugars^13,14^, organic acids^15^, alcohols^16^, and their mixtures. The nature of enzyme-based reactions allows EFCs to function at physiological pH, ambient pressure and room temperature. However, some enzymes operate at 85 °C and pH as low as 2.0^17,18^. Also, enzymes having redox nature offer brilliant specificity towards the fuels, hence lead to the development of membrane-less fuel cells resulting in the miniaturization of EFCs^19,20^.

The key element of EFCs is the enzymatic reaction in which electrons generated are transferred at the electrode surface. Because of the structure and size of the enzymes, the electron transfer mechanism between the redox-active site of enzymes and the electrode surface is specific. Generally, the electron transfer process between enzymes active sites and electrodes surface are of two types: direct electron transfer (DET) and mediated electron transfer (MET)^21,22^. In MET process redox-active materials, like methyl viologen, 2,2′-azino-bis(3-ethylbenzothiazoline-6-sulfonic acid) (ABTS) and ferrocene are used as mediators to carry the electrons from enzyme to electrode^23–25^. In this manner, the redox-active enzyme catalyzes the oxidation and reduction of co-substrate (mediator) with the regeneration of the mediator on the surface of the electrode. The employment of small and low molecular weight redox mediators that need low over potential is preferred, as they can permit fast electron transfer rates between enzyme and electrode surfaces with minimum power losses. However, this research work is focused on MET. The enzyme utilized here is glucose oxidase. Its redox-active site is deeply buried inside its protein pockets. Hence, a small redox mediator ferritin (Frt) was utilized that shuttled the electron from the deep-seated redox-active sites of GOx. Frt is an iron-cored globular-shaped protein having a diameter of 12 nm. Previous studies on Frt have shown that the protein shell may provide electron conductivity due to the mineral core^26–32^. Frt has approximately 4500 iron atoms, concentrated in the nanocavity, provides one-electron transfer per iron atom which is chemically reversible. The most important reason for the employment of Frt as the mediator is its redox potential which is quite close to the redox potential of GOx^33^.

This study utilizes the solution of chitosan as the medium of dispersion of RGO-PAni composite to enhance the lifespan of the immobilized GOx based bioanode in storage conditions. Highly deacylated chitosan (>85%) was preferred as it offers the finest properties for the implantable glucose-based EFCs. Undeniably, CHI with a high degree of deacylation is more stable, biocompatible, non-toxic, cheap, and less prone to degradation^34,35^. The less vulnerability to degradation reduces the inflammatory response of the host when CHI based composites are implanted. Unlike, the inflammatory response can be observed for the fast degradation of CHI causing the formation of amino saccharide. Moreover, the high degree of deacylation enhances the antimicrobial activity of the CHI^36^. The antimicrobial activity of the deacylated CHI in response to the pathogens was described *in-vivo* at pH 7.0^37^. This feature of the deacylated CHI minimizes the risk of infections in case any contamination happens while the implantation. Furthermore, CHI is an ideal biopolymer for the synthesis of a composite as it provides multiple functional moieties on its backbone for an easy covalent or physically adsorbed attachment of the conducting composite material.

Polyaniline (PAni) is among the most researched conducting polymer on account of its facile synthesis, inexpensive, high stability of the environment, adjustable conductivity via doping, presence of functional moieties, and electrochemical stability^38–46^. But its potential applications are limited because of its poor solubility in most of the organic solvents and inferior mechanical properties^47–51^. These problems have been minimized in various ways. For example, PAni-CHI composites have been explored for several applications such as sensors and biofuel cells^49,50^. The composites of PAni with CHI are proved to improve the stability, mechanical properties and other important properties of the conducting polymers^52–54^. Though, these improvements are achieved at the cost of good electrical conductivity.

At the same time, the integration of reduced graphene oxide (rGO) in PAni has also gained the attention of many scientists^55–57^. This is owing to the likeness in a conjugated electronic structure and intrinsic electroactivity of both the materials. Besides, rGO possesses a large surface area, good flexibility, high mechanical strength, and outstanding electrical conductivity. The sheet-like structure of rGO offers it as an ideal filler for polymers. The functional groups in rGO like carboxyl, hydroxyl, epoxy, and carbonyl support its distribution in a matrix of the polymer through π-π stacking, electrostatic forces, and hydrogen bonding interfacial interactions. The small amount of rGO has the potential to enhance the whole properties of its composite material. rGO-PAni composites have been employed for corrosion protection, supercapacitors, sensors, and biofuel cells^58–64^.

**Figure 1:**
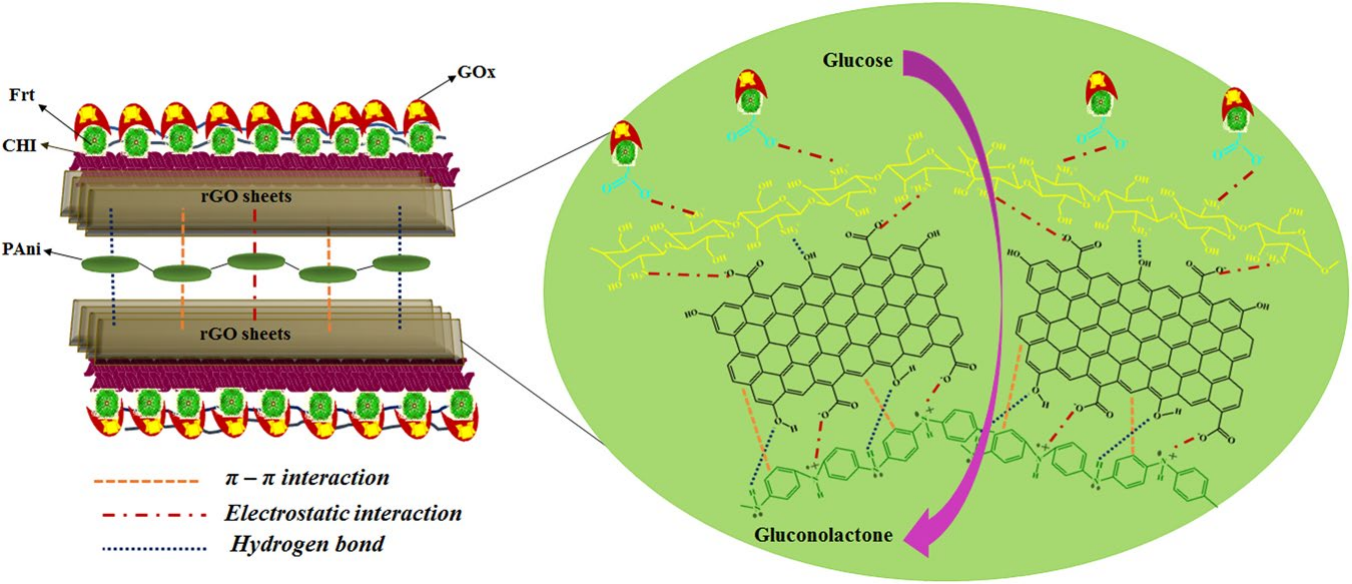
Schematic presentation of interaction among various component of the composite with the attachment of Frt and GOx.

In this research, a ternary composite was synthesized by grafting rGO-PAni on CHI. The useful features of CHI, PAni, and rGO were combined in the ternary composite to the already studied CHI-PAni, CHI-rGO and rGO-PAni^65–69^. Therefore, the excellent individual properties of CHI, rGO, and PAni such as biocompatibility, biodegradability, electrical conductivity, electrochemical activity, mechanical strength, friendly microenvironment to the enzyme, and large surface area to volume ratio are all considered in CHI@rGO-PAni ternary composite which can be potentially utilized for EFCs application as shown in Fig. 1.

## Materials and Method

### Materials

Hydrazine hydrate (N_2_H_4_.H_2_O), sulfuric acid (H_2_SO_4_), ammonium peroxy-di-sulfate (APS), 5-sulfosalicylic acid (SSA), and potassium permanganate (KMnO_4_) were received from Merck chemicals, India. Sodium nitrate (NaNO_3_), hydrogen peroxide (H_2_O_2_), aniline and ortho-phosphoric acid (H_3_PO_4_) were procured from Fisher Scientific, India. Ferritin (10 mg ml^−1^ in 0.15 M sodium chloride from horse spleen), and glutaraldehyde (Glu) were purchased from Sigma-Aldrich, India. Phosphate buffer saline (PBS) pH 7.0 and 5.0, GOx having activity from 100,000 to 150,000 units g^−1^ protein supplied by Central Drug House, India. Glucose was purchased from Himedia and Chitosan with a deacylation degree >90% was received from SRL, India. Double distilled water (DDW) was utilized throughout the investigations. All the materials were of analytical grade and used as received without further purification.

### Instrumentation

The voltammetric experiments and electrochemical impedance spectroscopy (EIS) were performed to check the electrochemical performances of the developed electrodes by using a computer controlled Potentiostat/Galvanostat (PGSTAT 302N Autolab) under nitrogen purging. The cell assembly consists of a conventional three-electrode system using a platinum wire electrode as a counter electrode (CE), KCl saturated Ag/AgCl reference electrode (RE) and glassy carbon electrode (GC) modified with CHI@rGO-PAni/Frt/GOx as a working electrode (WE) having a 0.07 cm^2^ surface area. The electrochemical impedance spectroscopy (EIS) experiments were recorded in a 0.1 M potassium chloride solution comprising of 2.0 mM K_4_[Fe(CN)_6_] in a frequency range from 0.1 to 10 kHz. The ultrasonicator was used for the partial cleaning of the electrode with ethanol. The morphological and chemical characterizations for the nanocomposite were carried out by FEI Quanta 250 FEG scanning electron microscope (SEM) and Fourier transform infrared (FTIR) spectroscopy recorded by Bruker (Platinum ATR-QL diamond system) in the spectral range from 4000 to 500 cm^−1^. All the experiments were conducted at room temperature.

### Preparation of the composite material

#### rGO synthesis

The graphene oxide (GO) was synthesized by Hummer’s method^70^. The obtained GO was reduced by NH_2_NH_2_.H_2_O^56^. A 1.0 g of GO was ultrasonicated in 100 mL of DDW to obtain a homogeneous suspension. To this suspension, NH_2_NH_2_.H_2_O was added drop by drop for 1 h while stirring it continuously and kept it at 90 °C for 4 h. The resulting mixture was filtered and rinsed several times with double distilled water to obtain rGO which is further dehydrated in the oven at 60 °C for 14 h^71^.

### Synthesis of rGO-PAni composite through *in-situ* polymerization

A 1.4 g of 5-sulfosalicylic acid (dopant) was dissolved in 200 mL water. A 0.5 g of rGO and 2 mL of aniline were added in it. The solution obtained was continuously agitated until a black homogeneous dispersion was formed which is further placed on an ice bath by maintaining the temperature below 6 °C. A solution of APS (5.02 g) was prepared in 100 mL of water by vigorous stirring. This solution was added dropwise into the mixture of rGO and aniline. After some time, the resulting solution turned into blackish-green in color, which is an indication of the formation of emeraldine salt of polyaniline. The precipitate obtained was carefully washed with EtOH and H_2_O constantly until a clear solution was obtained. The precipitate was dried at 50 °C^71,72^.

### CHI@rGO-PAni synthesis

A solution was prepared by dissolving 0.5 g of CHI in 50 mL of aqueous CH_3_COOH (2% v/v) by stirring it for 8 h. In that, rGO-PAni was dispersed by ultrasonication for 60 min. Figure 1 displays the mechanism of the composite formation in which the rGO sheets interact with the SSA doped PAni. The interaction of rGO and doped PAni occurred via hydrogen bond between phenolic OH and PAni radical (protonated by SSA). These interactions clutch the rGO sheets and PAni matrix together. However, the stronger bonds are the ones formed by electrostatic interactions originating from protonated N and lone pairs on OH group. The π-π stacking between the PAni and rGO rings further stabilizes the complex structure of the composite. Further, the rGO-PAni composite interacts with the protonated CHI via hydrogen bonding and electrostatic interaction that makes the huge structure of ternary composite stable.

### Preparation of the bioanode

The CHI@rGO-PAni/Frt/GOx bioelectrode was constructed as follows; first, the well-polished glassy carbon (GC) electrode was ultrasonicated for 10 min and rinsed with double distilled water. Further, the surface of the electrodes was cleaned by CV in 1.0 M sulfuric acid at a 50 mVs^−1^ potential sweep rate via the Autolab (PGSTAT 302N, Metrohm). The CHI@rGO-PAni dispersion was loaded on four GC electrodes with an amount of 4.0, 6.0, 8.0, and 10.0 μL. The CVs were taken for the sake of optimization. It was found that the electrode with 8.0 μL offered a better background current. The CHI@rGO-PAni modified three GC electrodes were altered with different amounts of Frt (2.0, 4.0, and 6.0 μL). It was observed from a CV curve that the electrode with 4.0 μL was performing better among the three modified electrodes. In the same manner, GOx solution (10.0 mg mL^−1^ GOx in 0.1 M sodium phosphate buffer saline of pH 5.0) was optimized for 4, 8, and 10 μL on the three GC electrodes with the pre-optimized materials. The CHI@rGO-PAni/Frt electrode loaded with 10 μL of GOx solution performed well than the other altered electrodes. Finally, the optimized GC/CHI@rGO-PAni/Frt/GOx bioanode was dried at room temperature. A 2.0 μL of 2% glutaraldehyde aqueous solution was drop cast to stabilize and crosslinked the CHI@rGO-PAni nanomaterial with Frt and GOx on the electrode surface.

## Results and Discussions

### Scanning electron microscope (SEM) analysis

**Figure 2:**
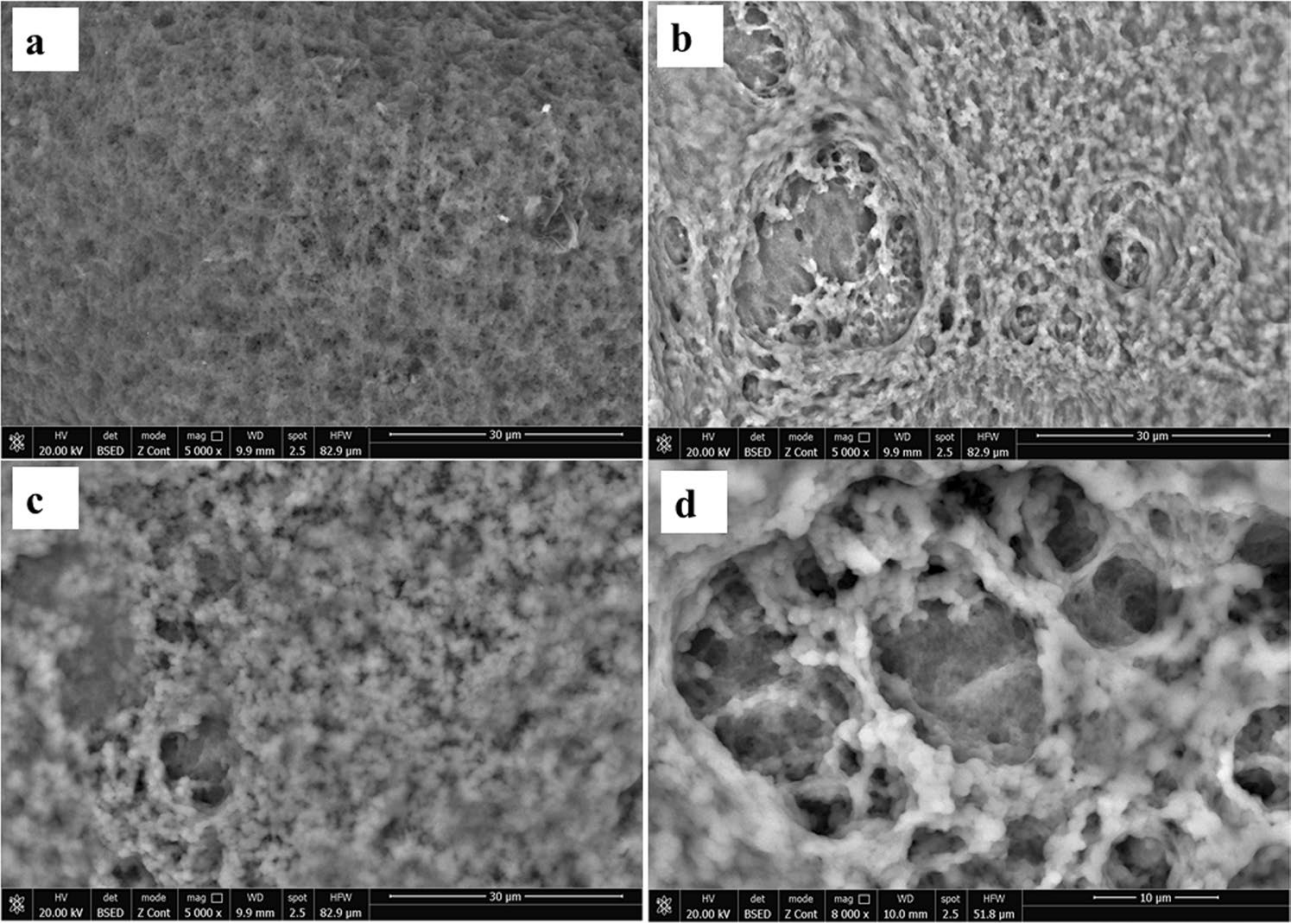
SEM images of (**a**) rGO-PAni, (**b**) CHI@rGO-PAni, (**c**,**d**) CHI@rGO-PAni/Frt/GOx.

The scanning electron microscopic morphologies of rGO-PAni, CHI@rGO-PAni, and CHI@rGO-PAni/Frt/GOx composites are displayed in Fig. 2(a–d). A fibrillar matrix of short granular structures can be seen in Fig. 2a. However, in the case of CHI@rGO-PAni, a granular matrix having an uneven distribution of pores can be seen which confirmed the disappearance of fibrous features when rGO-PAni was ultrasonicated in the solution of CHI. Interestingly, a porous structure can be observed in Fig. 2b, which is quite advantageous for the loading of mediator Frt with the immobilization of GOx. However, Fig. 2 c and d showed the successful capping of pores with the Frt and GOx. Besides, the porous structure allows the movement of the fuel towards the surface of the electrode for the redox reaction to occur.

### Fourier transform infrared (FTIR) analysis

**Figure 3:**
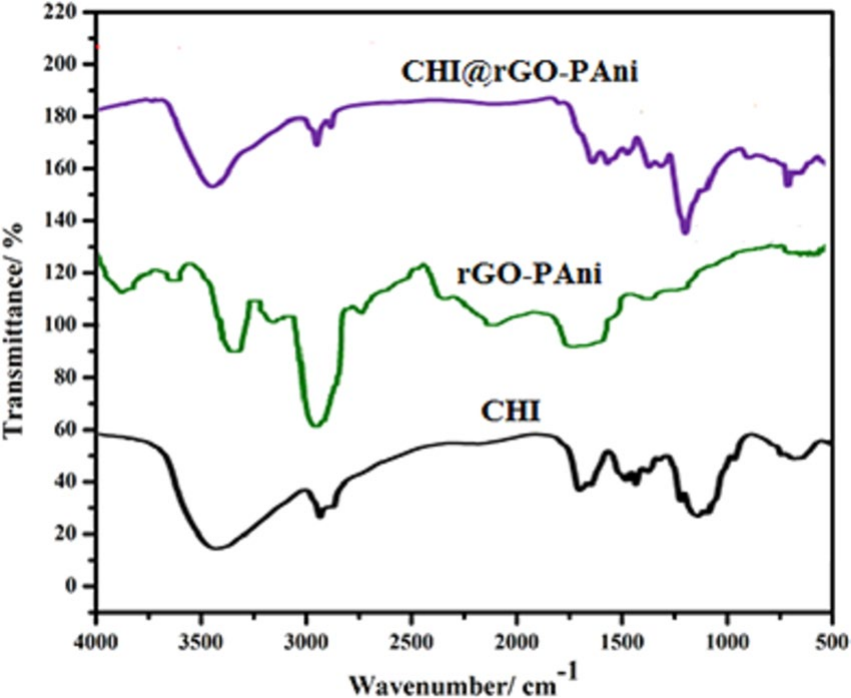
FTIR spectra of (**a**) CHI, (**b**) rGO-PAni, and (**c**) CHI@rGO-PAni.

The FTIR spectra of CHI, rGO-PAni and CHI@rGO-PAni composites are exhibited in Fig. 3. The important peaks of these composites are mentioned in Table 1. The intense broad peak at 3425 cm^−1^ is attributed to the overlying of O-H from SO_3_H with N-H characteristic stretching^35,61,62^. The significant peaks of PAni because of C = C ring stretching vibration of quinoid and benzoid in rGO-PAni and CHI@rGO-PAni could be found at 1487, 1491 cm^−1^ and 1571, 1584 cm^−1^, respectively. The peaks at 2853 and 2920 cm^−1^ in rGO-PAni and peaks at 2852 and 2918 cm^−1^ in CHI@rGO-PAni are due to C-H stretching. All the characteristic peaks of PAni-rGO and CHI are visible in the FTIR spectrum of a ternary composite. Though, the little shift was observed which might be due to the hydrogen bond among the components of the ternary composite^73^. Furthermore, the peaks found at 1008, 10028 and 1008, 1032 cm^−1^ in the spectra of rGO-PAni and CHI@rGO-PAni might be due to the presence of rGO in both of the composites.

**Table 1:** FTIR peaks of CHI, rGO-PAni and CHI@rGO-PAni.

Assignment	Absorption frequencies (cm^−1^)		
	CHI	rGO-PAni	CHI@rGO-PAni
N-H	1571	3425	3425
O-H	3423	3425	3425
C = C quinoid	—	1487	1484
C = C benzoid	—	1567	1584
C-H	2921, 2877	2825, 2920	2852,2918
C = O	1667	—	1629
NH_2_	3423	—	
C-O-C	1152	—	1158

### Electrochemical activity of prepared electrodes

**Figure 4:**
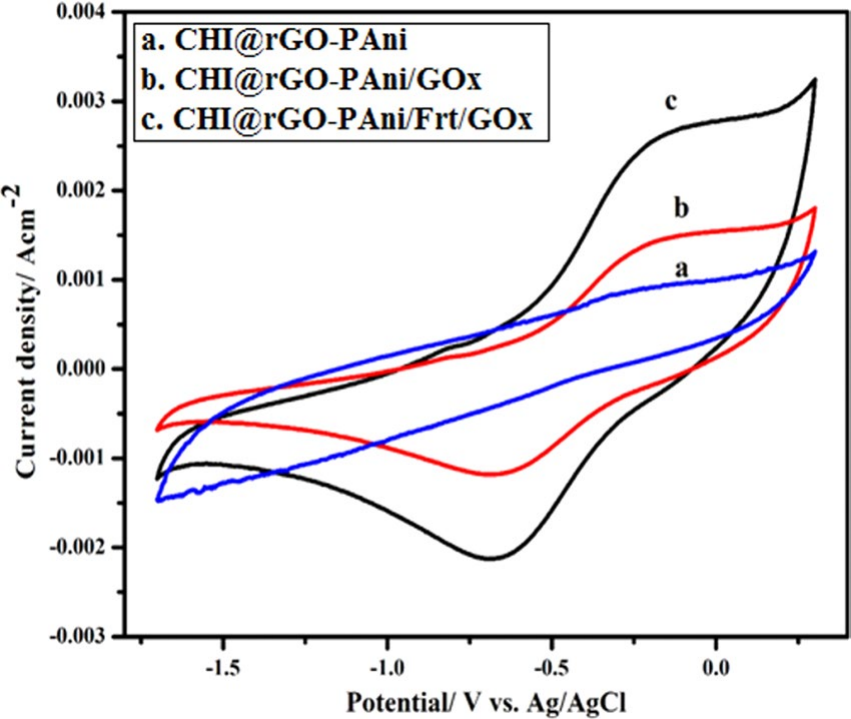
Cyclic voltammograms of (**a**) CHI@rGO-PAni, (**b**) CHI@rGO-PAni/GOx, and (**c**) CHI@rGO-PAni/Frt/GOx in 0.1 M PBS of pH 7.0 at a sweep rate of 100 mVs^−1^.

Figure 4 exhibits the cyclic voltammograms (CVs) of (a) CHI@rGO-PAni, (b) CHI@rGO-PAni/GOx, and (c) CHI@rGO-PAni/Frt/GOx at a potential sweep rate of 100 mVs^−1^. No obvious redox peaks were noticed on the curve (a) in PBS (pH = 7.0), but the curve (b) shows high background current in comparison to the curve corresponding to CHI@rGO-PAni electrode because of large electroactive surface of the rGO-PAni nanocomposite grafted on CHI immobilized with GOx. The experiment recorded for CHI@rGO-PAni/Frt/GOx shows that the current density increases by almost two times compared with the CHI@rGO-PAni/GOx bioelectrode. This indicates that the efficiency of electron movement from deeply buried redox cofactor (∼13 Å) within the GOx to the conducting support CHI@rGO-PAni was enhanced by the mediator Frt. As the previous chemical and electrochemical researches on Frt have shown that the proteinaceous casing may behave as an electron conductor and mineralized core enhances the electronic conductivity of protein^26,27^. A pair of quasi-reversible redox peaks were observed on curves (b) and (c). These results indicated that the CHI@rGO-PAni/Frt/GOx ternary bio-nanocomposite might have the electrocatalytic activity towards the reduction of GOx cofactor FAD to FADH_2_^74^.

**Figure 5:**
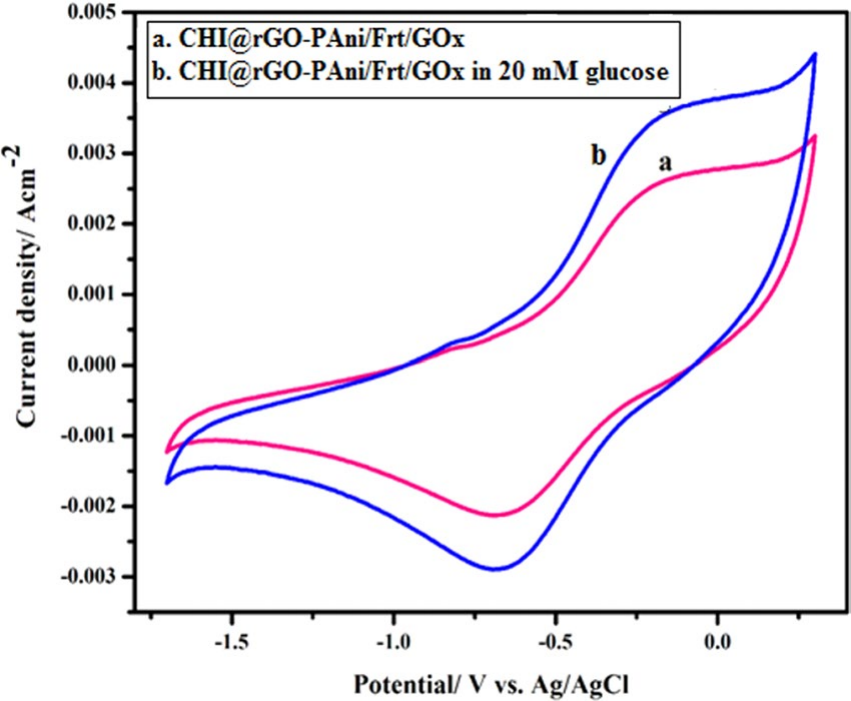
Cyclic voltammograms of (**a**) CHI@rGO-PAni/Frt/GOx in PBS of pH 7.0, (**b**) CHI@rGO-PAni/Frt/GOx in PBS of pH 7.0 with 20 mM glucose.

Figure 5 reveals the bioelectrocatalytic activity of the CHI@rGO-PAni/Frt/GOx in 0.1 M PBS with (20 mM glucose) and without glucose in the potential window of −1.7 to −0.7. The CV of the CHI@rGO-PAni/Frt/GOx bio-nanocomposite exhibited a significant catalytic redox function with the generation of anodic current i.e. 2.5 ± 0.04 mA cm^−2^, which is relatively more than the current produced by the CHI@rGO-PAni composite material. However, the adding of glucose in PBS triggered the much magnification in the current response up to 3.5 ± 0.02 mA cm^−2^ accompanied by the appearance of obvious quasi-reversible oxidoreduction peaks in the CV of the CHI@rGO-PAni/Frt/GOx. The pair of redox peaks appeared due to the conversion of the cofactor of GOx, i.e., FAD/FADH_2_, while the conversion of glucose to gluconolactone. This suggests that the redox reaction occurring at the modified electrode surface was intervened with the redox mediator Frt, and the required conducting platform was offered by the conducting CHI@rGO-PAni nanocomposite. Despite, with the help of Frt, the conducting properties and the suitable capacitive behavior of the nanocomposite concurrently brought the efficient electron transfer from the profoundly seated redox-active cofactor of GOx at the interface of electrode and electrolyte^33,46,75^.

The clear redox peaks with an apparent formal peak potential (average of anodic and cathodic peak potentials) at −0.45 V was calculated, which is near to the standard electrode potential of FAD/FADH_2_ (−0.50 V) at pH 7.0^76^. This indicates the bioactivity of the GOx retained after immobilization on the CHI@rGO-PAni/Frt. The peak to peak separation (ΔE = E_pa_-E_pc_) at 0.428 V vs. Ag/AgCl reference electrode was observed at 100 mVs^−1^ for CHI@rGO-PAni/Frt/GOx in the presence of glucose. This indicated the fast-mediated electron transfer of GOx, owing to the uniform porous structure and large specific surface area of the CHI@rGO-PAni/Frt biocomposite. These findings demonstrate that the as-prepared nanocomposite assists the beneficial immobilization of the GOx because of the high surface area, excellent electrocatalytic activity, and the suitable microenvironment provided by the rGO-PAni grafted on CHI. Hence, it is confirmed that the fabricated bioanode is a potential candidate to be employed in the construction of glucose-based EFCs.

**Figure 6:**
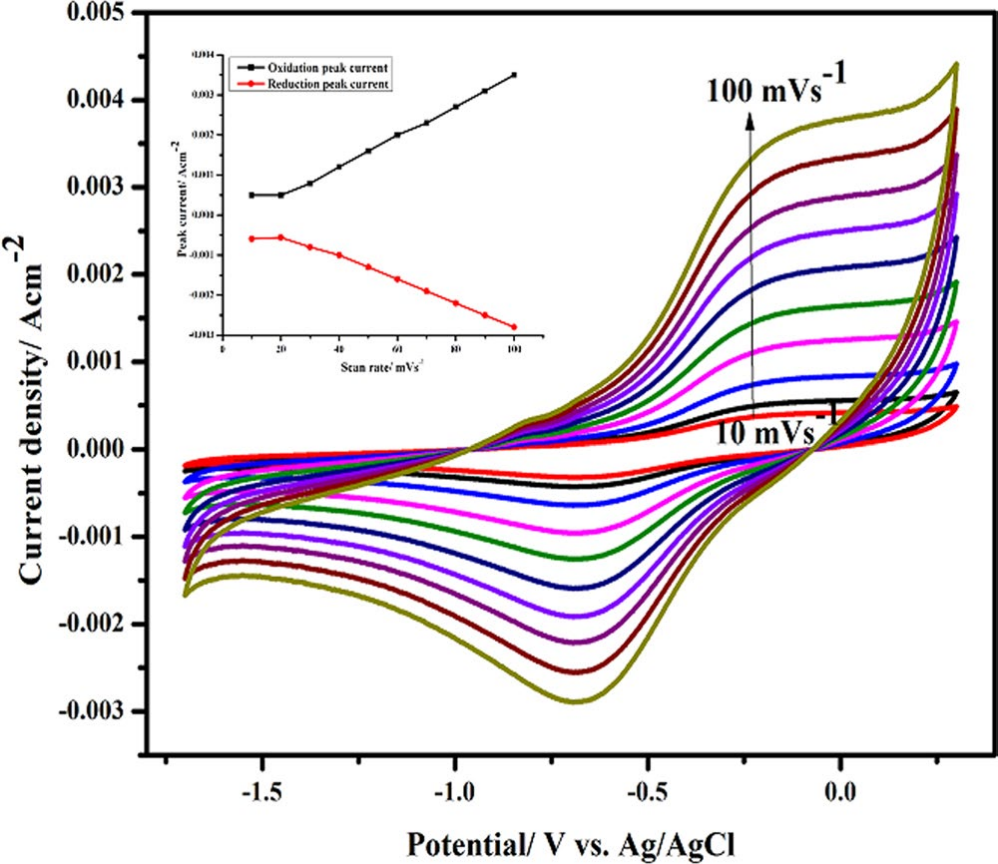
Cyclic voltammograms in 0.1 M PBS of pH 7.0 with 20 mM glucose at scan rate varying from 10 to 100 mVs^−1^ (from inner to outer); inset is the plot of peak current vs. scan rate.

The influence of various potential sweep rates on the cyclic voltammetric performance of CHI@rGO-PAni/Frt/GOx bioanode was recorded. Figure 6 shows that the response of peak currents gradually amplified with the increasing sweep rate along with the shifting of oxidation and reduction peaks in the right (positive) and left (negative) direction, respectively. Which assured the linear dependence of oxidation peak current (Ipa) and reduction peak current (Ipc) with linear regression equations I_pa_ = 0.00045 *v* – 0.0001, R^2^ = 0.99 and I_pc_ = −0.00032*v*– 0.0008, R^2^ = 0.98 at the scan rates (10–100 mVs^−1^), hence implying a quasi-reversible and surface-controlled electrochemical process^77,78^. The CVs in a broad spectrum of scan rates evident the significant electrochemical behavior of the developed bioanode.

The relation of redox peak potentials with the Napierian logarithm of the scan rates was analyzed to evaluate electrochemical parameters by utilizing the equations given below^79^:

$${E}_{Pa}={E}_{f}+\left[\frac{RT}{(1-\alpha )nF}\right]\mathrm{ln}\left[\frac{(1-\alpha )Fn}{RT{k}_{s}}v\right]$$

$${E}_{Pc}={E}_{{\rm{f}}}-\left[\frac{RT}{\alpha nF}\right]\mathrm{ln}\left[\frac{\alpha Fn}{RTks}v\right]$$

Where *E*_f_ is the formal potential, α is the charge transfer coefficient of the system, *v* is the scan rate, *n* is the number of electron transfer, *k*_s_ is the heterogeneous electron transfer rate constant, *T*, *R*, and *F* have their usual meanings.

**Figure 7:**
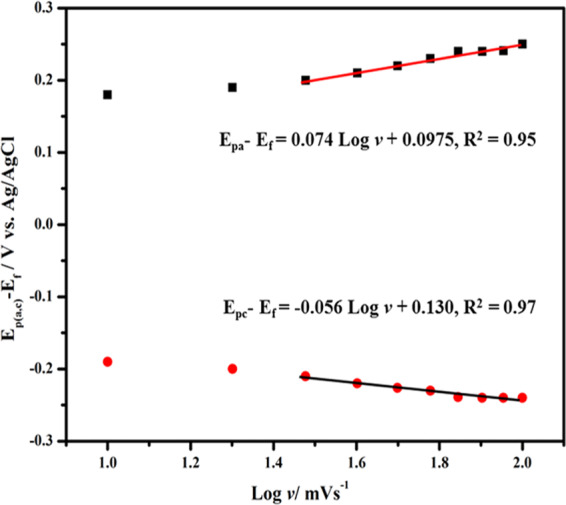
Reliance of oxidation (square) and reduction (circle) peak potentials on the logarithm of scan rate for GOx functionalized CHI@rGO-PAni/Frt electrode at various scan rates.

The n and α were calculated to be 1.88 and 0.50, respectively, from the slope of the lines of Fig. 7. The Laviron equation was applied to calculate the rate constant of heterogeneous electron transfer (*k*_s_ = 0.50 ± 0.01 s^−1^) of the CHI@rGO-PAni/Frt/GOx modified electrode^80^. The *k*_s_ of CHI@rGO-PAni/Frt/GOx was slightly lower than the similar work has reported which is due to the larger peak potential separation.

The surface coverage concentration of the bioelectroactive GOx, I^*^ is estimated from the integration of the charge of the anodic peak in the CV curve in accordance with the Faraday law, Q = nFAI^*^, where Q is the charge integrated from the cathodic peak, A is the effective surface area of the electrode in cm^2^, F is the Faraday constant, and I^*^ is the surface coverage of the enzyme GOx. I^*^ of GOx on CHI@rGO-PAni/Frt. It was calculated to be 3.80 × 10^−8^ mol cm^−2^, which is comparatively four times higher than the bare GC electrode (2.86 × 10^−12^ mol cm^−2^)^81^. This indicates saturated adsorption of GOx in CHI@rGO-PAni/Frt bionanocomposite.

### Electrochemical impedance spectroscopy (EIS)

**Figure 8:**
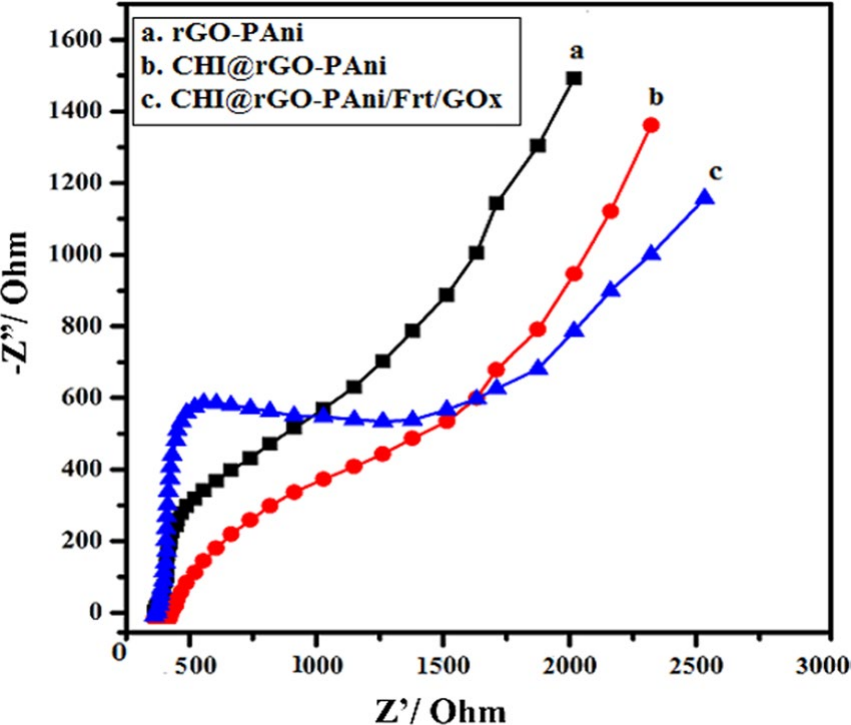
Nyquist plots of EIS for (**a**) rGO-PAni, (**b**) CHI@rGO-PAni, and (**c**) CHI@rGO-PAni/Frt/GOx.

EIS is a powerful technique for the investigation of impedance changes occuring during the redox reaction. The modified surface of the electrodes was characterized by EIS to analyze the electronic transfer properties of the composite materials at the interface of electrode and electrolyte^82^. This technique was used to confirm the interaction among rGO-PAni, CHI@rGO-PAni and CHI@rGO-PAni/Frt/GOx tailored electrodes. The Nyquist plots displayed a semi-circle region with different diameters and a linear region, as shown in Fig. 8. The semi-circle region observed at larger frequencies indicates the electron transfer limited process and the linear region at smaller frequencies designates the diffusion process. The resistance to electron transfer (Ret) at the surface of the electrode can be computed through the semi-circle diameter. The diameter of the semi-circle region decreased among the samples, with CHI@rGO-PAni/Frt/GOx > CHI@rGO-PAni > rGO-PAni. These results approve the idea that the nanocomposite can be employed to promote the electron transfer. The Ret of the rGO-PAni is lesser than the CHI@rGO-PAni, indicating the rGO-PAni grafted CHI could hinder the transfer of electron to some extent. When the ternary composite CHI@rGO-PAni was modified by Frt and GOx, Ret increased which indicated the successful adsorption of GOx and Frt.

### Linear sweep voltammetry (LSV) study

**Figure 9:**
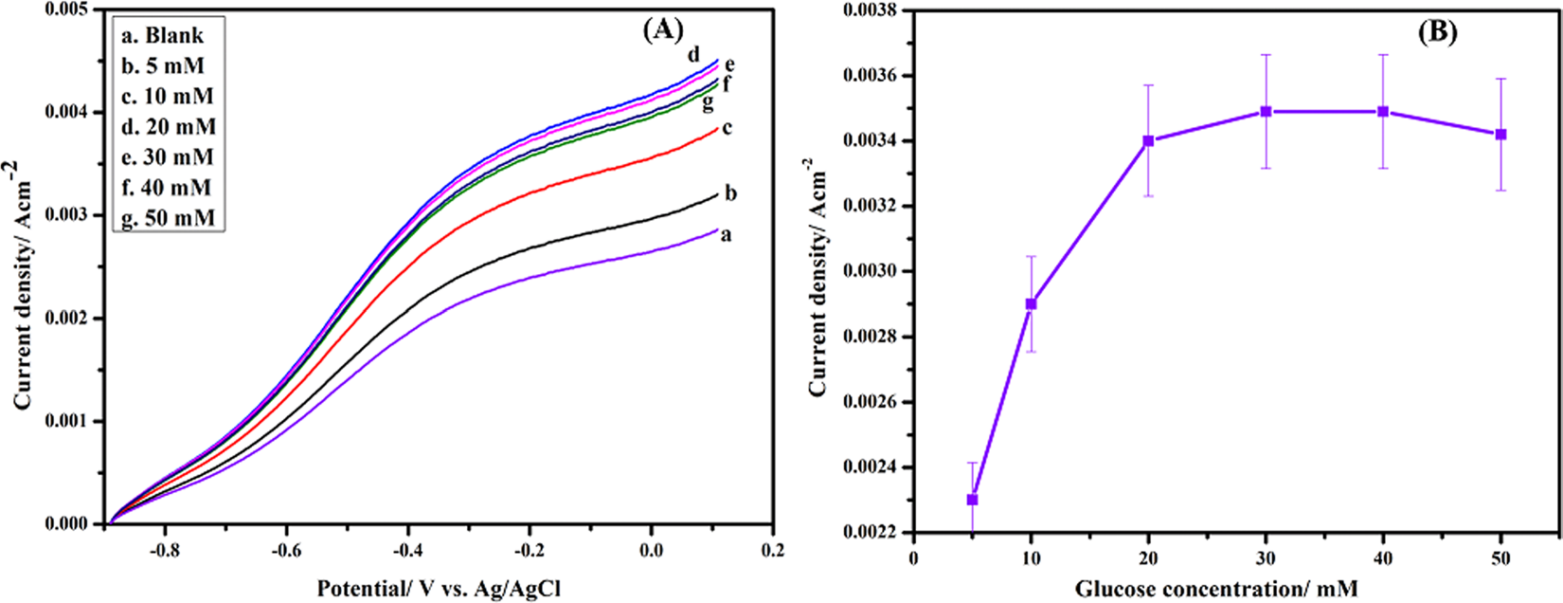
(**A**) LSV curves at various concentration of glucose in 0.1 M PBS of pH 7.0, (**B**) A plot of current response vs. glucose concentration.

The LSV investigation was conducted to study the bioelectrocatalytic oxidation response generated by the CHI@rGO-PAni/Frt/GOx modified bioanode relating to the various concentration of glucose in PBS of pH 7.0 (Fig. 9(A)). The electrocatalytic oxidation of glucose began at −0.46 V (E onset). The current density generated at anode raises with the addition in the glucose concentration upto 30 mM beyond which the current density got saturated because of the hindrance caused by the higher glucose concentration^29,30^. Hence, the catalytic current remains constant over time with the addition of more amount of glucose.

The current response obtained from the modified anode versus the various glucose concentrations is plotted in Fig. 9(B). The current density amplified from 2.3 ± 0.02 mA cm^−2^ for 5.0 mM glucose to 2.9 ± 0.02 mA cm^−2^ for 10 mM glucose and this then reached to the equilibrium at 3.5 ± 0.02 mA cm^−2^ for 20 mM glucose at 100 mVs^−1^. This demonstrates the appropriate biocatalytic oxidation of glucose on the GC/CHI@rGO-PAni/Frt/GOx electrode. The saturation in current density accomplished by the ternary nanocomposite modified electrode is comparable with the other previously tested anodes as shown in Table 2. Hence, this research broadens the outlook of the real-world application to develop enzyme-catalyzed high-performance EFCs.

**Table 2:** Comparison with the other similar studies employing CHI.

Electrode configuration	I^*^	Current density	Ref
GC/CHI/CNCs	6.24 × 10^−10^ mol cm^−2^	434 μAcm^−2^	^83^
CHI/graphene	1.12 × 10^−9^ molcm^−2^	_	^84^
ECFs/MWCNT/CHI/BOD	_	1.2 mA cm^−2^	^85^
CHI/MWCNT/ n [commo-3,3′-Fe-(closo-2,1-C_2_B_9_H_11_)_2_]^−^/GOx	_	1.24 mAcm^−2^	^86^
MWCNT/Fc/GOx/CHI	_	73 μA	^87–89^
CHI@rGO-PAni/Frt/GOx	3.8 × 10^−8^ molcm^−2^	3.5 mAcm^−2^	This work

### Stability

**Figure 10:**
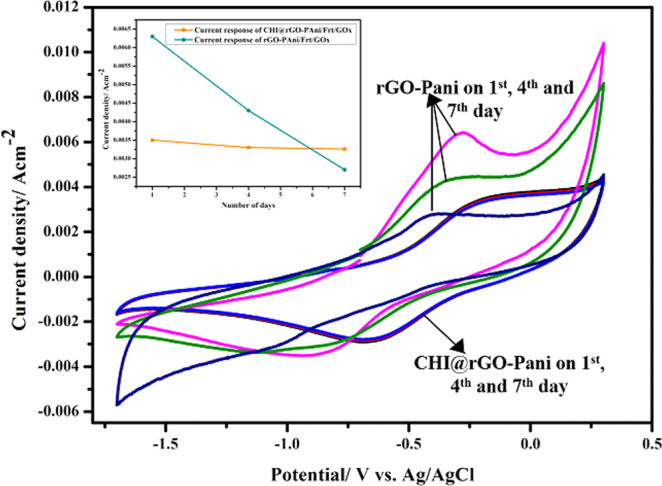
Cyclic voltammograms of stored CHI@rGO-PAni/Frt/GOx electrode at 1^st^, 4^th^, and 7^th^ day.

The stability of CHI@rGO-PAni/Frt/GOx and rGO-PAni/Frt/GOx bioanodes was also studied via electrochemical investigation. These two electrodes were stored at 4 °C and CVs were recorded periodically at 1^nd^, 4^th^ and 7^th^ day. It was observed that initially, the rGO-PAni/Frt/GOx bioanode provided higher current than CHI@rGO-PAni/Frt/GOx, but with time the current density falls rapidly for the same electrode, as shown in Fig. 10. However, CHI@rGO-PAni/Frt/GOx bioanode showed lesser current density but better storage stability after one week and it retained 95% of its initial current response. These results exhibited that the mediated electrochemistry of GOx loaded on the CHI@rGO-PAni/Frt has good stability owing to the properties endowed by the integration of rGO-PAni on CHI which provided mechanical durability to the nanocomposite. Besides, the high surface area of the nanocomposite offered a large number of active sites to hold more biocatalysts. Therefore, it is clear that CHI@rGO-PAni/Frt/GOx bioanode has significant electrochemical stability.

## Conclusion

The synthesized CHI@rGO-PAni biocomposite was used to construct a bioanode for glucose-based EFCs application showed good electrochemical properties along with substantial stability due to the collaborative effects among CHI, PAni, and rGO. The mediator Frt possessing the redox activity smoothed the movement of the electrons between the profoundly seated GOx active sites and the electrode surface. To ensure the mediated electrocatalytic activity, CV was performed and the resulting voltammograms suggest that the immobilized enzyme showed good bioelectrocatalytic activity for glucose oxidation. The prepared bioanode proved capable of generating a maximum current (3.5 ± 0.02 mAcm^−2^) for the oxidation of 20 mM glucose. The improvement of the performance was due to the porosity and large surface area of the anode material permitting higher loading of active enzymes and ease of fuel diffusion through the CHI@rGO-PAni customized electrode. The CHI@rGO-PAni/Frt/GOx bioanode showed lesser current density but better storage stability after one week and it retained 95% of its initial response. The involvement of CHI enriches the biocompatibility of the prepared electrode which is beneficial for implantable EFCs. This study explained the potential of the prepared anode for the possible development of improved EFC performance. Future work will focus on the maximum utilization of the CHI along with conducting material to enhance long-term stability accompanying high electron transfer efficiency.

## References

[1] Carrette L, Friedrich KA, Stimming U (2001). Fuel Cells - Fundamentals and Applications. Fuel Cells.

[2] Windmiller JR, Wang J (2013). Wearable Electrochemical sensors and biosensors: A review. Electroanalysis.

[3] Zhong Z (2018). A high-performance glucose/oxygen biofuel cell based on multi-walled carbon nanotube films with electrophoretic deposition. J. Electroanal. Chem..

[4] Katz E, MacVittie K (2013). Implanted biofuel cells operating *in vivo* – methods, applications and perspectives – feature article. Energy Environ. Sci..

[5] Fan S (2020). Controllable display of sequential enzymes on yeast surface with enhanced biocatalytic activity toward efficient enzymatic biofuel cells. J. Am. Chem. Soc..

[6] Rasmussen M, Abdellaoui S, Minteer SD (2016). Enzymatic biofuel cells: 30 years of critical advancements. Biosens. Bioelectron.

[7] Calabrese Barton S, Gallaway J, Atanassov P (2004). Enzymatic biofuel cells for implantable and microscale devices. Chem. Rev..

[8] Kim J, Jia H, Wang P (2006). Challenges in biocatalysis for enzyme-based biofuel cells. Biotechnol. Adv..

[9] Cracknell JA, Vincent KA, Armstrong FA (2008). Enzymes as working or inspirational electrocatalysts for fuel cells and electrolysis. Chem. Rev..

[10] Cosnier S, Gross J, Le Goff A, Holzinger M (2016). Recent advances on enzymatic glucose/oxygen and hydrogen/oxygen biofuel cells: Achievements and limitations. J. Power Sources.

[11] Cosnier S, Gross AJ, Giroud F, Holzinger M (2018). Beyond the hype surrounding biofuel cells: What’s the future of enzymatic fuel cells?. Curr. Opin. Electrochem.

[12] Xiao X (2019). Tackling the challenges of enzymatic (Bio)fuel cells. Chem. Rev..

[13] Kamitaka Y, Tsujimura S, Setoyama N, Kajino T, Kano K (2007). Fructose/dioxygen biofuel cell based on direct electron transfer-type bioelectrocatalysis. Phys. Chem. Chem. Phys..

[14] Hou C, Liu A (2017). An integrated device of enzymatic biofuel cells and supercapacitor for both efficient electric energy conversion and storage. Electrochim. Acta.

[15] Sakai K, Kitazumi Y, Shirai O, Takagi K, Kano K (2017). High-power formate/dioxygen biofuel cell based on mediated electron transfer type bioelectrocatalysis. ACS Catal.

[16] Ramanavicius A, Kausaite A, Ramanaviciene A (2008). Enzymatic biofuel cell based on anode and cathode powered by ethanol. Biosens. Bioelectron.

[17] Kontani A (2014). A bioanode using thermostable alcohol dehydrogenase for an ethanol biofuel cell operating at high temperatures. Electroanalysis.

[18] Wang X (2018). Electron transfer in an acidophilic bacterium: interaction between a diheme cytochrome and a cupredoxin. Chem. Sci.

[19] Zhang L (2012). Small-size biofuel cell on paper. Biosens. Bioelectron.

[20] Feng R (2016). Rational design of xylose dehydrogenase for improved thermostability and its application in development of efficient enzymatic biofuel cell. Enzyme Microb. Technol..

[21] Ghindilis AL, Atanasov P, Wilkins E (1997). Enzyme-catalyzed direct electron transfer: Fundamentals and analytical applications. Electroanalysis.

[22] Falk M, Blum Z, Shleev S (2012). Direct electron transfer based enzymatic fuel cells. Electrochim. Acta.

[23] Kano K, Ikeda T (2000). Fundamentals and practices of mediated bioelectrocatalysis. Anal. Sci..

[24] Nasar A, Perveen R (2019). Applications of enzymatic biofuel cells in bioelectronic devices – A review. Int. J. Hydrogen Energy.

[25] Umar MF, Nasar A (2018). Reduced graphene oxide/polypyrrole/nitrate reductase deposited glassy carbon electrode (GCE/RGO/PPy/NR): Biosensor for the detection of nitrate in wastewater. Appl. Water Sci.

[26] Axford DN, Davis JJ (2007). Electron flux through apo-and holoferritin. Nanotechnology.

[27] Xu D, Watt GD, Harb JN, Davis RC (2005). Electrical conductivity of ferritin proteins by conductive AFM. Nano Lett..

[28] Haque, S. U., Nasar, A., Inamuddin & Asiri, A. M. Preparation and characterization of a bioanode (GC/MnO_2_/PSS/Gph/Frt/GOx) for biofuel cell application. *Int. J. Hydrogen Energy* **44**, 7308–7319 (2019).

[29] Haque SU (2017). Optimization of glucose powered biofuel cell anode developed by polyaniline-silver as electron transfer enhancer and ferritin as biocompatible redox mediator. Sci. Rep.

[30] Inamuddin, Haque SU, Naushad M (2016). Electrochemical studies of biocatalytic anode of sulfonated graphene/ferritin/glucose oxidase layer-by-layer biocomposite films for mediated electron transfer. Enzyme Microb. Technol..

[31] Perveen R, Nasar A, Inamuddin, Asiri AM, Mishra AK (2018). Optimization of MnO_2_-graphene/polythioaniline (MnO_2_-G/PTA) hybrid nanocomposite for the application of biofuel cell bioanode. Int. J. Hydrogen Energy.

[32] Perveen, R., Nasar, A., Inamuddin, Kanchi, S. & Kashmery, H. A. Development of a ternerry condunting composite (PPy/Au/CNT@Fe_3_O_4_) immobilized FRT/GOD bioanode for glucose/oxygen biofuel cell applications. *Int. J. Hydrogen Energy* (2020) 10.1016/j.ijhydene.2020.02.175.

[33] Shin HJ (2011). Electrocatalytic characteristics of electrodes based on ferritin/carbon nanotube composites for biofuel cells. Sensors Actuators B Chem.

[34] Pillai CKS, Paul W, Sharma CP (2009). Chitin and chitosan polymers: Chemistry, solubility and fiber formation. Prog. Polym. Sci..

[35] El Ichi S (2014). Chitosan improves stability of carbon nanotube biocathodes for glucose biofuel cells. Chem. Commun..

[36] Dash M, Chiellini F, Ottenbrite RM, Chiellini E (2011). Chitosan—A versatile semi-synthetic polymer in biomedical applications. Prog. Polym. Sci..

[37] Jeon SJ, Oh M, Yeo W-S, Galvão KN, Jeong KC (2014). Underlying mechanism of antimicrobial activity of chitosan microparticles and implications for the treatment of infectious diseases. PLoS One.

[38] Janaki V (2012). Application of bacterial extracellular polysaccharides/polyaniline composite for the treatment of Remazol effluent. Carbohydr. Polym.

[39] Huang WS, MacDiarmid AG (1993). Optical properties of polyaniline. Polymer (Guildf).

[40] Borah R, Banerjee S, Kumar A (2014). Surface functionalization effects on structural, conformational, and optical properties of polyaniline nanofibers. Synth. Met.

[41] Fratoddi I, Venditti I, Cametti C, Russo MV (2015). Chemiresistive polyaniline-based gas sensors: A mini review. Sensors Actuators B Chem.

[42] Qiu S (2017). Long-term corrosion protection of mild steel by epoxy coating containing self-doped polyaniline nanofiber. Synth. Met.

[43] Kohl M, Kalendová A (2015). Effect of polyaniline salts on the mechanical and corrosion properties of organic protective coatings. Prog. Org. Coatings.

[44] Kumar L, Rawal I, Kaur A, Annapoorni S (2017). Flexible room temperature ammonia sensor based on polyaniline. Sensors Actuators B Chem.

[45] Zhang K, Luo J, Yu N, Gu M, Sun X (2019). Synthesis and excellent electromagnetic absorption properties of reduced graphene oxide/PANI/BaNd_0.2_Sm0.2Fe11.6O19 nanocomposites. J. Alloys Compd..

[46] Haque SU, Inamuddin, Nasar A, Asiri AM (2018). Fabrication and characterization of electrochemically prepared bioanode (polyaniline/ferritin/glucose oxidase) for biofuel cell application. Chem. Phys. Lett..

[47] Liao G, Li Q, Xu Z (2019). The chemical modification of polyaniline with enhanced properties: A review. Prog. Org. Coatings.

[48] Su N (2015). Improving electrical conductivity, thermal stability, and solubility of polyaniline-polypyrrole nanocomposite by doping with anionic spherical polyelectrolyte brushes. Nanoscale Res. Lett..

[49] Cao Y, Smith P, Heeger AJ (1993). Counter-ion induced processibility of conducting polyaniline. Synth. Met.

[50] Zhou X (2012). A renewable bamboo carbon/polyaniline composite for a high-performance supercapacitor electrode material. J. Solid State Electrochem..

[51] Cao Y, Qiu J, Smith P (1995). Effect of solvents and co-solvents on the processibility of polyaniline: I. solubility and conductivity studies. Synth. Met.

[52] Thanpitcha T, Sirivat A, Jamieson AM, Rujiravanit R (2006). Preparation and characterization of polyaniline/chitosan blend film. Carbohydr. Polym.

[53] Tiwari A, Singh V (2007). Synthesis and characterization of electrical conducting chitosan-graft-polyaniline. Express Polym. Lett.

[54] Shukla SK, Tiwari A (2011). Synthesis of chemical responsive chitosan–grafted-polyaniline bio-composite. Adv. Mater. Res.

[55] Luo J, Jiang S, Wu Y, Chen M, Liu X (2012). Synthesis of stable aqueous dispersion of graphene/polyaniline composite mediated by polystyrene sulfonic acid. J. Polym. Sci. Part A Polym. Chem.

[56] Nguyen VH, Lamiel C, Kharismadewi D, Tran VC, Shim J-J (2015). Covalently bonded reduced graphene oxide/polyaniline composite for electrochemical sensors and capacitors. J. Electroanal. Chem..

[57] Chen N (2017). *In situ* one-pot preparation of reduced graphene oxide/polyaniline composite for high-performance electrochemical capacitors. Appl. Surf. Sci..

[58] Kumar NA (2012). Polyaniline-grafted reduced graphene oxide for efficient electrochemical supercapacitors. ACS Nano.

[59] Stankovich S (2006). Graphene-based composite materials. Nature.

[60] Li R, Liu L, Yang F (2013). Preparation of polyaniline/reduced graphene oxide nanocomposite and its application in adsorption of aqueous Hg(II). Chem. Eng. J.

[61] Chang C-H (2012). Novel anticorrosion coatings prepared from polyaniline/graphene composites. Carbon N. Y.

[62] Jafari Y, Ghoreishi SM, Shabani-Nooshabadi M (2016). Polyaniline/graphene nanocomposite coatings on copper: Electropolymerization, characterization, and evaluation of corrosion protection performance. Synth. Met.

[63] Al-Mashat L (2010). Graphene/polyaniline nanocomposite for hydrogen sensing. J. Phys. Chem. C.

[64] Perveen R, Inamuddin, Nasar A, Beenish, Asiri AM (2018). Synthesis and characterization of a novel electron conducting biocomposite as biofuel cell anode. Int. J. Biol. Macromol..

[65] Wang X, Bai H, Yao Z, Liu A, Shi G (2010). Electrically conductive and mechanically strong biomimetic chitosan/reduced graphene oxide composite films. J. Mater. Chem..

[66] Fan L, Luo C, Sun M, Li X, Qiu H (2013). Highly selective adsorption of lead ions by water-dispersible magnetic chitosan/graphene oxide composites. Colloids Surfaces B Biointerfaces.

[67] Travlou NA, Kyzas GZ, Lazaridis NK, Deliyanni EA (2013). Graphite oxide/chitosan composite for reactive dye removal. Chem. Eng. J.

[68] Jia Z-R (2018). Effects of filler loading and surface modification on electrical and thermal properties of epoxy/montmorillonite composite. Chinese Phys. B.

[69] Wu H, Wu G, Ren Y, Li X, Wang L (2016). Multishelled metal oxide hollow spheres: Easy synthesis and formation mechanism. Chem. - A Eur. J.

[70] Hummers WS, Offeman RE (1958). Preparation of graphitic oxide. J. Am. Chem. Soc..

[71] Usman F (2019). Synthesis and characterisation of a ternary composite of polyaniline, reduced graphene-oxide and chitosan with reduced optical band gap and stable aqueous dispersibility. Results Phys.

[72] Mitra M (2015). Reduced graphene oxide-polyaniline composites—synthesis, characterization and optimization for thermoelectric applications. RSC Adv.

[73] Kabiri R, Namazi H (2016). Synthesis of cellulose/reduced graphene oxide/polyaniline nanocomposite and its properties. Int. J. Polym. Mater. Polym. Biomater..

[74] Heller A (2004). Miniature biofuel cells. Phys. Chem. Chem. Phys..

[75] Perveen R (2017). Electrocatalytic performance of chemically synthesized PIn-Au-SGO composite toward mediated biofuel cell anode. Sci. Rep.

[76] Dai ZH, Ni J, Huang XH, Lu GF, Bao JC (2007). Direct electrochemistry of glucose oxidase immobilized on a hexagonal mesoporous silica-MCM-41 matrix. Bioelectrochemistry.

[77] Shan C (2009). Direct electrochemistry of glucose oxidase and biosensing for glucose based on graphene. Anal. Chem..

[78] Zhao X, Mai Z, Kang X, Zou X (2008). Direct electrochemistry and electrocatalysis of horseradish peroxidase based on clay–chitosan-gold nanoparticle nanocomposite. Biosens. Bioelectron.

[79] Campbell AS (2015). Membrane/mediator-free rechargeable enzymatic biofuel cell utilizing graphene/single-wall carbon nanotube cogel electrodes. ACS Appl. Mater. Interfaces.

[80] Laviron E (1974). Surface linear potential sweep voltammetry. J. Electroanal. Chem. Interfacial Electrochem.

[81] Liu S, Ju H (2003). Reagentless glucose biosensor based on direct electron transfer of glucose oxidase immobilized on colloidal gold modified carbon paste electrode. Biosens. Bioelectron.

[82] Ehret R (1997). Monitoring of cellular behaviour by impedance measurements on interdigitated electrode structures. Biosens. Bioelectron.

[83] Kang Z (2017). Direct electrochemistry and bioelectrocatalysis of glucose oxidase in CS/CNC film and its application in glucose biosensing and biofuel cells. RSC Adv.

[84] Kang X (2009). Glucose Oxidase–graphene–chitosan modified electrode for direct electrochemistry and glucose sensing. Biosens. Bioelectron.

[85] Engel AB (2017). Optimization of chitosan film-templated biocathode for enzymatic oxygen reduction in glucose hybrid biofuel cell. J. Electrochem. Soc..

[86] Buckner SW, Jelliss PA, Nukic A, Zalocusky ER, Schumacher J (2010). A metallacarborane redox mediator for an enzyme-immobilized chitosan-modified bioanode. Bioelectrochemistry.

[87] Park, H. J. *et al*. Fabrication of CNT/ferrocene/glucose oxidase/chitosan-layered bioanode for glucose/oxygen biofuel cells. *Mol. Cryst. Liq. Cryst*. **539**, 238/[578]–246/[586] (2011).

[88] Solonaru AM, Grigoras M (2017). Water-soluble polyaniline/graphene composites as materials for energy storage applications. Express Polym. Lett.

[89] Patil SL, Chougule MA, Pawar SG, Sen S, Patil VB (2012). Effect of Camphor Sulfonic Acid Doping on Structural, Morphological, Optical and Electrical Transport Properties on Polyaniline-ZnO Nanocomposites. Soft Nanosci. Lett.

